# Transfer learning classification of suspicious lesions on breast ultrasound: is there room to avoid biopsies of benign lesions?

**DOI:** 10.1186/s41747-024-00480-y

**Published:** 2024-10-28

**Authors:** Paolo De Marco, Valerio Ricciardi, Marta Montesano, Enrico Cassano, Daniela Origgi

**Affiliations:** 1 Medical Physics Unit, IEO European Institute of Oncology IRCCS, Milan, Italy; 2 Medical Physics School, University of Milan, Milan, Italy; 3 Breast Imaging Division, Radiology Department, IEO European Institute of Oncology IRCCS, Milan, Italy

**Keywords:** Artificial intelligence, Breast neoplasms, Machine learning, Neural networks (computer), Ultrasound

## Abstract

**Background:**

Breast cancer (BC) is the most common malignancy in women and the second cause of cancer death. In recent years, there has been a strong development in artificial intelligence (AI) applications in medical imaging for several tasks. Our aim was to evaluate the potential of transfer learning with convolutional neural networks (CNNs) in discriminating suspicious breast lesions on ultrasound images.

**Methods:**

Transfer learning performances of five different CNNs (Inception V3, Xception, Densenet121, VGG 16, and ResNet50) were evaluated on a public and on an institutional dataset (526 and 392 images, respectively), customizing the top layers for the specific task. Institutional images were contoured by an expert radiologist and processed to feed the CNNs for training and testing. Postimaging biopsies were used as a reference standard for classification. The area under the receiver operating curve (AUROC) was used to assess diagnostic performance.

**Results:**

Networks performed very well on the public dataset (AUROC 0.938–0.996). The direct generalization to the institutional dataset resulted in lower performances (max AUROC 0.676); however, when tested on BI-RADS 3 and BI-RADS 5 only, results were improved (max AUROC 0.792). Good results were achieved on the institutional dataset (AUROC 0.759–0.818) and, when selecting a threshold of 2% for classification, a sensitivity of 0.983 was obtained for three of five CNNs, with the potential to spare biopsy in 15.3%–18.6% of patients.

**Conclusion:**

In conclusion, transfer learning with CNNs may achieve high sensitivity and might be used as a support tool in managing suspicious breast lesions on ultrasound images.

**Relevance statement:**

Transfer learning is a powerful technique to exploit the performances of well-trained CNNs for image classification. In a clinical scenario, it might be useful for the management of suspicious breast lesions on breast ultrasound, potentially sparing biopsy in a non-negligible number of patients.

**Key Points:**

- Properly trained CNNs with transfer learning are highly effective in differentiating benign and malignant lesions on breast ultrasound.
- Setting clinical thresholds increased sensitivity.
- CNNs might be useful as support tools in managing suspicious lesions on breast ultrasound.

**Graphical Abstract:**

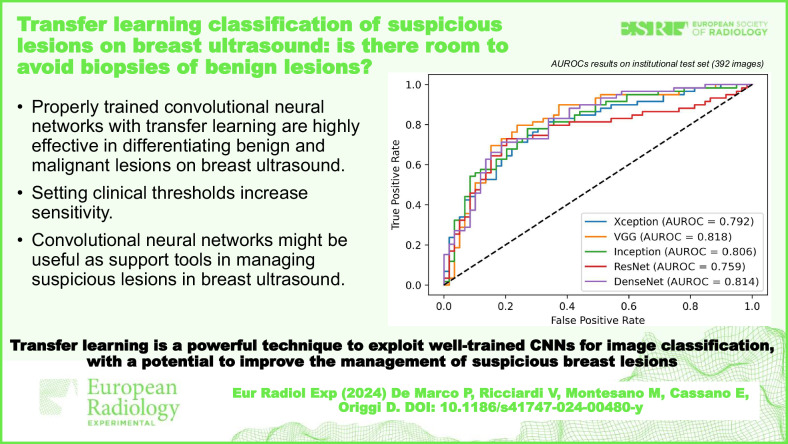

## Background

Breast cancer (BC) is the most common malignancy in women and the second leading cause of cancer death, so early diagnosis of BC remains crucial [1]. Mammography is the first-line examination for BC screening while ultrasound is recommended as a first-line examination in young women, during pregnancy or breastfeeding, and as an additional examination in women with dense breasts after mammography [2]. Thus, ultrasound is considered the most important adjunct method in clinical detection and diagnosis of BC for its high availability, cost-effectiveness, acceptable diagnostic performance, and non-invasive and real-time capabilities [3].

However, ultrasound remains an operator-dependent examination, and interpreting breast ultrasound is a challenging task. Radiologists evaluate images using different features including lesion size, shape, margin, echogenicity, and orientation which vary significantly across patients. Ultimately, they determine if the imaged findings are benign, need short-term follow-up imaging, or require a biopsy based on their suspicion of malignancy, according to the Breast Imaging Reporting and Data System (BI-RADS®) [4]. Such recommendations carry considerable intra-reader variability and breast ultrasound has been criticized for increasing the number of false positive (FP) findings [5, 6]. In fact, after breast ultrasound, 5–15% of patients are recalled and 4–8% of these undergo biopsy for FP results [2].

For these reasons, in recent years, there has been a strong development of artificial intelligence (AI) to be applied to breast ultrasound, covering three steps: image processing, image segmentation, and feature extraction.

In particular, AI is capable of extracting quantitative information (features) from images and using them for classification tasks. Such features can be hand-crafted and subsequently handled by machine learning algorithms that create prediction models and decision support tools, or directly extrapolated from images.

Deep learning algorithms, in particular convolutional neural networks (CNNs), have been recognized as a reliable approach to learning predictive features directly from original images [7]. Many CNN models have been developed for object detection and classification, such as ResNet50 [8], InceptionV3 [9], Xception [10], VGG16 [11], and DenseNet121 [12]. Such networks employ different architectures and strategies in order to improve the efficiency of computation, speed, and accuracy of classification. Comprehensive description of the involved networks is beyond the scope of the study however the interested reader can find more details in the literature [8–12].

Although deep CNNs have been shown to be efficient classifiers, they always require a large amount of training data, which retrieval can be a challenging task for medical imaging data. To overcome this limitation, transfer learning is believed to be a powerful tool for training deeper networks without overfitting [13, 14].

Studies on transfer learning have shown that features learned from significantly large image sets are highly transferable to a variety of image recognition tasks [13].

There are basically two approaches to transferring knowledge from one model to another: one can be described as a “feature extractor”, and the other one as “fine-tuning”.

In the first approach, the last layer of the previously trained model is replaced with one or more custom-built layers, so only the parameters in the top layer(s) are trained for the new task, whereas all other parameters remain frozen. In this way, the previously trained model acts as a feature extractor that collects information about the images in input while the decision process relies only on the last layer(s), specially designed for the classification task. In cases when data for training are limited, such as in medical settings, the “feature extractor” approach might be the only option to train a model without overfitting, because of the limited number of parameters to train, present only in the custom layer(s). On the other hand, when more data are available for training, it is possible to transfer all parameters and train up to the entire network, performing the “fine-tuning” approach. Even if, in principle, to initialize all weights randomly, using those of the pre-trained model helps in convergence and in the final performances of the networks, also in the case of a large amount of data available [13].

A common approach is to use both the “feature extractor” and the “fine-tuning”: at first, the pre-trained model is fully frozen, and only parameters of the custom top layer(s) are updated in the training process until convergence. After that, it is possible to unfreeze some of the last layers to update parameters trying to achieve better performances. Such kind of approach should be carefully evaluated, because the higher the number of parameters to train, the higher the probability of overfitting with loss of robustness and generalizability of the model [13].

The main advantages of transfer learning include reducing training time, providing better performance for neural networks, and requiring limited data. Usually, networks trained on a very large number of images have early layers that are very similar (*i.e.*, layers that perform the same tasks): for example, CNNs tend to learn edges, textures, and patterns in the first layers, and these layers capture the features that are broadly useful for analyzing the natural images. Such layers can be considered as generic feature extractors and can be used in many different types of settings. The closer we get to the output, the more specific features the layers tend to learn, that is why in fine-tuning only the last layers are eventually unfreeze [14].

Transfer learning has been used for lesion classification, with pre-trained models from which authors could build a metamodel [15], extract transfer features for subsequent classification [16, 17], slightly modify the CNN [18] or create a fine-tuned model from the saved features of the pre-trained model [19].

In this work, a more simple and straightforward approach is pursued, as described later, to evaluate the potential clinical application of transfer learning with well-established pre-trained CNNs and a limited number of images in discriminating suspicious breast lesions on ultrasound images.

## Methods

### Image datasets

Two datasets were used: the public breast ultrasound images (BUSI) dataset [20], containing 350 benign and 176 malignant images, each with its mask, and an institutional dataset, containing 392 images of suspicious consecutive lesions acquired from January 14, 2020 to March 21, 2021. Institutional images were acquired using an RS80A ultrasound machine coupled with a linear probe LA4-18B (Samsung, Seul, Republic of Korea), and the images chosen for reporting were then contoured by an expert radiologist in order to obtain masks for segmentation. For both datasets, images with measurements or annotations were excluded from the analysis. BI-RADS scores were collected from radiological reports and results of pathological anatomy examinations after breast biopsy were considered as gold standard to classify the lesions. It is worth noticing that images from the BUSI dataset had only the associated label (benign or malignant) without further information such as clinical status or BI-RADS score.

### Preprocessing

**Fig. 1:**
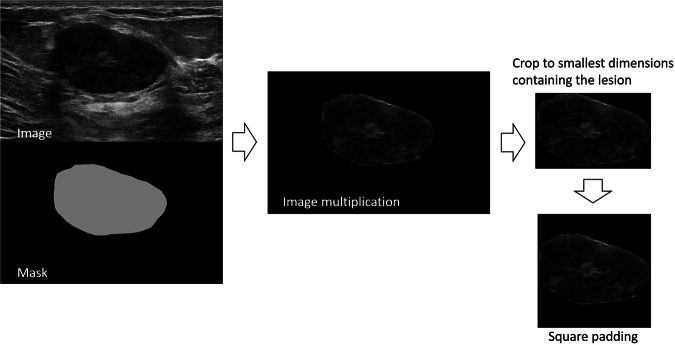
Pipeline of the image preprocessing applied before training the CNNs

In order to feed the CNNs some preprocessing was needed to obtain correct images as input. Every image was multiplied by its segmentation mask (*i.e.*, contour performed by an expert breast radiologist, M.M., with more than 5 years of experience), and the result was cropped to get the minimum area containing the lesion. Images were then zero-padded (*i.e.*, rows or columns of zeros were added) to obtain square images, necessary to feed the CNNs: The pipeline of image preprocessing is shown in Fig. 1. All tasks were performed through a Python script specifically designed to automate the process.

### Study design

At first, we performed training and test on the BUSI dataset, in order to set a benchmark for the proposed approach. Later, we further validated the models built with the BUSI dataset only, in order to evaluate their generalizability to the institutional data and, in the end, we evaluated performances of the networks on institutional data only.

When dealing with BUSI and institutional images, 70% (*n* = 368 and 264 for BUSI and institutional dataset, respectively) of the dataset was used for training, and 30% (*n* = 158 and 118 for BUSI and institutional, respectively) for testing, maintaining the proportion of benign and malignant cases in both groups. Five different networks (InceptionV3 [9], Xception [10], ResNet50 [8], VGG16 [11], and DenseNet121 [12]) were used as base models, and then trained and tested on the two datasets.

Pretrained models were loaded starting from the weights from ImageNet [21], original top layers were removed and replaced by the following configuration:

- Top dense layer with “relu” activation.
- Dropout layer (dropout rate = 50%).
- Fully connected layer for classification with the “sigmoid” activation function.

Transfer learning training was performed with the following parameters:

- Loss function = binary cross-entropy.
- Batch size = 64.
- ADAM optimizer learning rate = 10^-4^ with momentum = 0.9.
- Early stopping with patience = 10.
- Epochs 150 (maximum).

Data were augmented with flipping, random zoom up to 20%, and a combination of the two, for each image. Network performances were evaluated in terms of sensitivity, specificity, accuracy, F1-score, and area under the receiver operating curve (AUROC). CNNs were fed by square images composed of 224 × 224 pixels as input, and a probability of malignancy was returned as output for each image. A classification threshold was used to populate the two classes (benign or malignant) with values above or below the selected probability (the default was 0.5, so at first a lesion with the probability of malignancy below 0.5 was classified as benign).

Training, validation, and testing were performed with a Python script specifically designed for this task, using Keras [22] and TensorFlow [23] libraries.

### Metrics

The metrics used to evaluate the performances of the different CNNs are reported below.

Sensitivity, *i.e.*, the proportion of correctly classified positive image patches:

$${Sensitivity}\,=\,\frac{{{TP}}}{{{TP}}\,+\,{{FN}}}\,$$

where TP is true positive and FN is false negative.

Specificity, *i.e.*, the proportion of correctly classified negative image patches:

$${{Specificity}}\,=\,\frac{{{TN}}}{{{TN}}\,+\,{{FP}}}$$

where TN is true negative and FP.

Accuracy, *i.e.*, the overall number of correctly classified instances

$${{Accuracy}}\,=\,\frac{{{TP}}\,+\,{{TN}}}{{{TP}}\,+\,{{TN}}\,+\,{{FP}}\,+\,{{FN}}}$$

Positive predictive value (PPV), *i.e.*, the proportion of TP over the total classified as positive

$${{PPV}}\,=\,\frac{{{TP}}}{{{TP}}\,+\,{{FP}}}$$

F1-score, which combines the impact of sensitivity and using the harmonic mean, giving equal penalties to extreme values:

$${{F1}}\, {{score}}\,=\,2\,\times\,\frac{{{Sensitivity}}\,\times\,{{PPV}}}{{{Sensitivity}}\,+\,{{PPV}}}$$

AUROC (already above-mentioned), which represents the ability of a binary classifier to distinguish between classes and is used as a summary of the model’s performance. The higher the AUC, the better the model’s performance at distinguishing between the positive and negative classes.

In addition, sensitivity and specificity at different thresholds were computed in order to evaluate possible applications in clinical scenarios. Lastly, predictions of the best-performing networks were averaged to assess added value in discriminating lesions.

## Results

Of the 392 institutional images, 196 were benign and 196 malignant. The benign lesions were: fibroadenoma (*n* = 111); cyst (*n* = 29); adenoma (*n* = 13); mastitis (*n* = 12); ductal hyperplasia (*n* = 10); and other findings (*n* = 13). The malignant lesions were: invasive ductal carcinoma (*n* = 144); lobular carcinoma (*n* = 32); mucinous carcinoma (*n* = 4); apocrine carcinoma (*n* = 2); cribriform carcinoma (*n* = 1); and ductal carcinoma *in situ* (*n* = 9).

The BI-RADS classification was as follows: 99 BI-RADS 3, 207 BI-RADS 4 (77 4a, 28 4b, and 102 4c), and 69 BI-RADS 5, while information was not retrieved for 17 cases. Relative frequencies of benign and malignant cases as a function of BI-RADS classification are shown in Table 1.

**Table 1:** Absolute and relative frequencies of benign and malign lesions for different BI-RADS classifications in the institutional dataset

BI-RADS	Benign	Malignant	Total
3	99 (100.0%)	0 (0.0%)	99 (100.0%)
4a	70 (90.9%)	7 (9.1%)	77 (100.0%)
4b	13 (46.4%)	15 (53.6%)	28 (100.0%)
4c	5 (4.9%)	97 (95.1%)	102 (100.0%)
5	0 (0.0%)	69 (100.0%)	69 (100.0%)
Unknown	9	8	17 (100%)
Total	196 (50.0%)	196 (50.0%)	392 (100.0%)

**Fig. 2:**
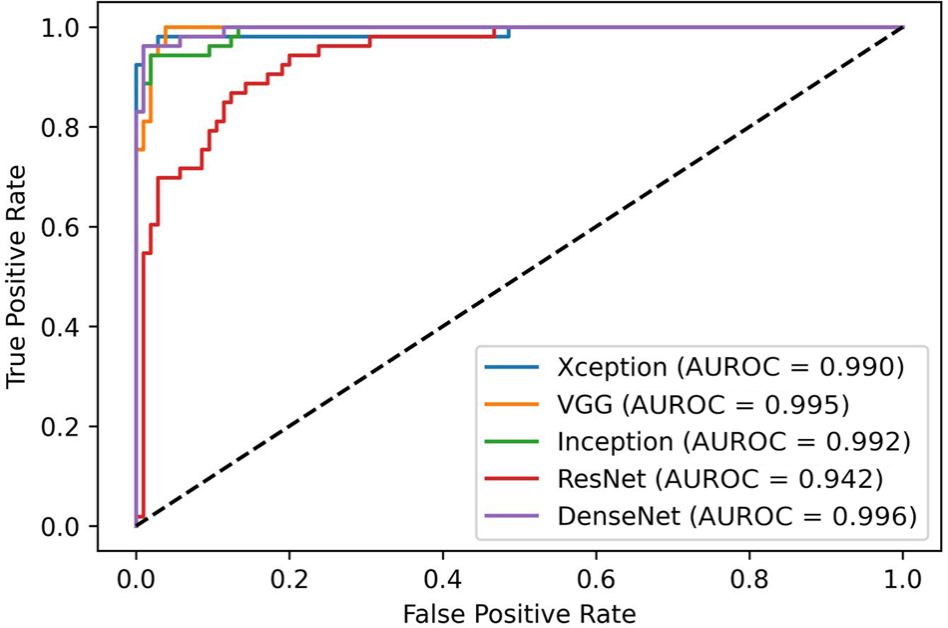
Results on BUSI test set. *AUROC,* Area under the receiver operating curve

Performances for the BUSI dataset are shown in Table 2 (with all metrics computed on the test subset with a default threshold of 50%), with performances across all networks showing AUROCs in the range from 0.938 to 0.996. DenseNet121 showed the highest sensitivity and specificity (0.962 and 0.990, respectively), while ResNet50 had the lowest results, with low sensitivity (0.558) and lowest AUROC (0.938). Visual representation of AUROCs is shown in Fig. 2.

**Table 2:** Performances on the BUSI dataset

CNN	Sensitivity	Specificity	Accuracy	F1-score	AUC
InceptionV3	0.943	0.981	0.968	0.952	0.992
Xception	0.962	0.971	0.968	0.953	0.990
VGG16	0.906	0.981	0.956	0.932	0.995
ResNet50	0.588	0.973	0.846	0.717	0.938
DenseNet121	0.962	0.990	0.981	0.971	0.996

*CNN* Convolutional neural networks

**Fig. 3:**
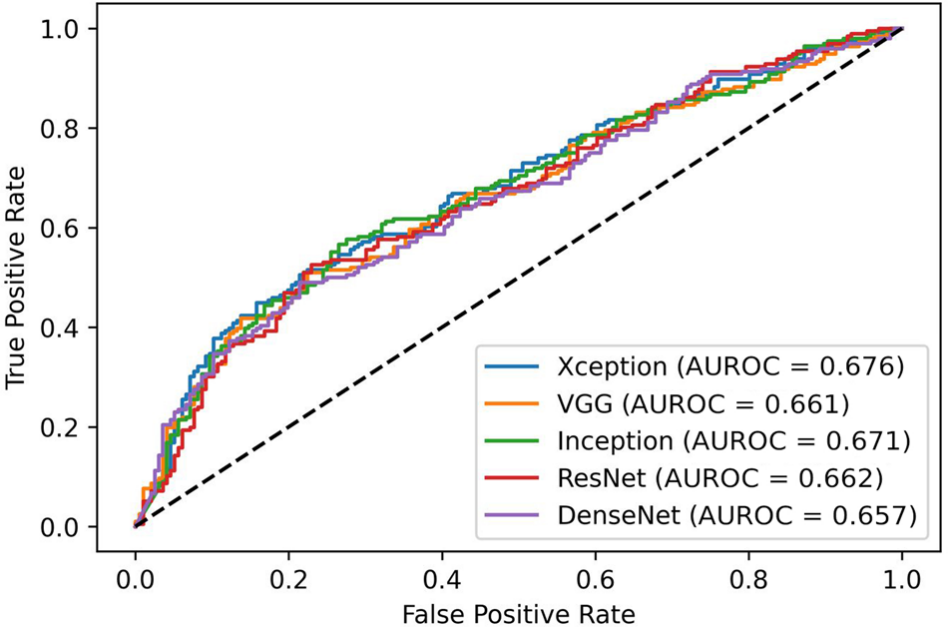
Validation of BUSI models on institutional images. *AUROC*, Area under the receiver operating curve

**Fig. 4:**
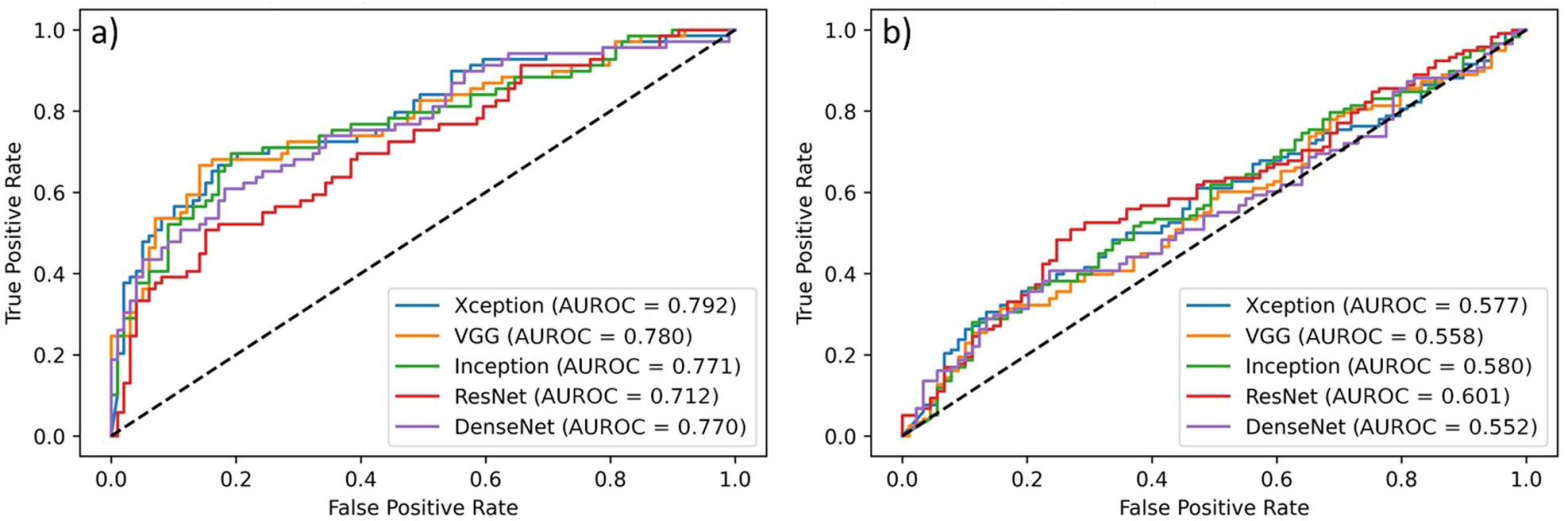
Validation of BUSI models on BI-RADS 3 and 5 (**a**) and on BI-RADS 4 subsets (**b**). *AUROC*, Area under the receiver operating curve

Despite such good performances, when trying to generalize to the institutional dataset the models built with the BUSI images, we obtained worse results, as shown in Fig. 3. Investigating those results, we considered the validation according to different BI-RADS classifications, and we found that the performances on a dataset containing only BI-RADS 3 and BI-RADS 5 images were significantly higher than those obtained on a BI-RADS 4 dataset, as shown in Fig. 4a, b.

To further emphasize the loss of performances when BUSI-trained models are validated against BI-RADS 3 and 5 or against BI-RADS 4, sensitivity and specificity are presented in Table 3.

**Table 3:** Performances for the institutional validation according to BI-RADS classification

CNN	BI-RADS 3 and BI-RADS 5		BI-RADS 4	
	Sensitivity	Specificity	Sensitivity	Specificity
InceptionV3	0.696	0.798	0.475	0.663
Xception	0.652	0.838	0.398	0.753
VGG16	0.536	0.929	0.322	0.787
ResNet50	0.246	0.970	0.186	0.899
DenseNet121	0.551	0.828	0.398	0.764

*CNN* Convolutional neural networks

**Fig. 5:**
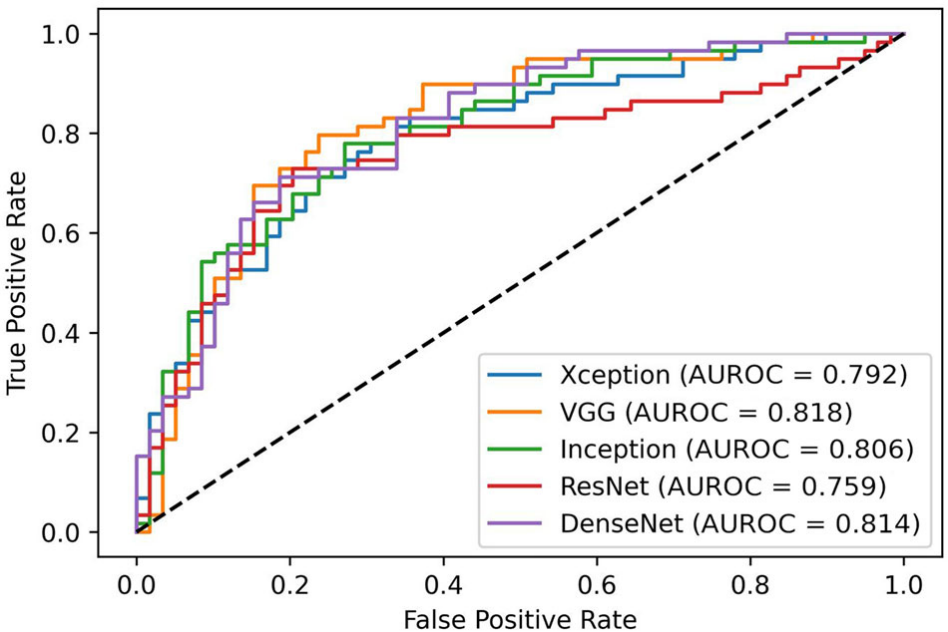
Results on the institutional test set. *AUROC*, Area under the receiver operating curve

CNN performance on our institutional dataset is shown in Table 4 (with all metrics computed on the test subset with a default threshold of 50%). All CNNs show a similar level of accuracy, with the ResNet50 having a different trend, with the lowest sensitivity but the highest specificity. The corresponding AUROCs are shown in Fig. 5.

**Table 4:** CNNs performances on the institutional dataset

CNN	Sensitivity	Specificity	Accuracy	F1-score	AUC
InceptionV3	0.847	0.559	0.703	0.741	0.806
Xception	0.797	0.661	0.729	0.746	0.792
VGG16	0.847	0.627	0.737	0.763	0.818
ResNet50	0.542	0.864	0.703	0.646	0.759
DenseNet121	0.898	0.525	0.712	0.757	0.814

*CNN* Convolutional neural networks

**Fig. 6:**
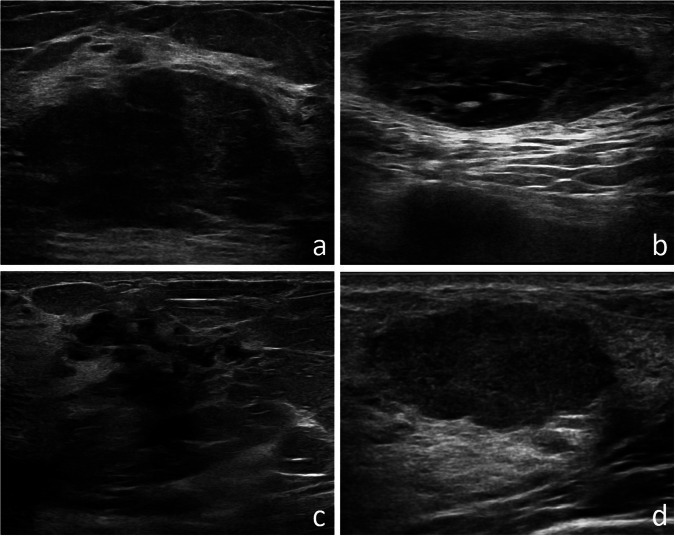
Examples of cases classified as FN (**a**, **b**) and FP (**c**, **d**) by the CNNs when using the 2% threshold. **a** Oval and hypoechoic lesion with circumscribed margins, finally pathologically diagnosed as invasive ductal carcinoma. **b** Oval lesion with circumscribed margins and heterogeneous echo pattern finally pathologically diagnosed as invasive ductal carcinoma. **c** Irregular lesion with non-well-circumscribed margins and heterogeneous echo pattern, finally pathologically diagnosed as fibrocystic disease. **d** Oval and hypoechoic lesion with polilobulated margins finally pathologically diagnosed as fibroadenoma

The results at different threshold values are shown in Table 5: it is worth noticing that decreasing the threshold (*i.e.*, the value above which a lesion is considered malignant) increased sensitivity at a cost of specificity. With 2% threshold, in particular, InceptionV3, Xception, and DenseNet121 had a sensitivity of 0.983, and specificity ranging from 0.153 to 0.186. Examples of FN and FP cases are shown in Fig. 6. In addition, when averaging the predictions of these three best-performing networks, we achieved full sensitivity (1.000) with 0.102 specificity.

**Table 5:** Performances on the institutional dataset at different thresholds

	Threshold 1%		Threshold 2%		Threshold 10%	
CNN	Sensitivity	Specificity	Sensitivity	Specificity	Sensitivity	Specificity
InceptionV3	0.983	0.101	0.983	0.169	0.949	0.356
Xception	0.983	0.119	0.983	0.186	0.915	0.305
VGG16	1.000	0.000	1.000	0.017	0.983	0.220
ResNet50	1.000	0.000	1.000	0.000	1.000	0.000
DenseNet121	1.000	0.119	0.983	0.153	0.966	0.271

*CNN* Convolutional neural networks

## Discussion

The aim of our study was to evaluate the performances of pre-trained models in order to classify suspicious breast lesions on ultrasound images, exploiting the possibility for clinical application. At first, we show that overall excellent results were obtained in classifying lesions from the BUSI dataset.

Our performance was higher than those obtained by Ali et al [15] who found an accuracy of 0.83 for Inception V3, and 0.84 for DenseNet121, while we found 0.968, and 0.981, respectively. Conversely, their result was higher for ResNet50, with 0.88 accuracy compared to our 0.846. This is possibly due to the different numbers of epochs for training, which could have led to suboptimal performances, since in their work the maximum was set at 30, compared to ours at 150, with early stopping and restoration of best weights.

Pathan et al [24] obtained a 0.92 accuracy on the BUSI dataset with a multi-head CNN, showing that with customized built CNNs it is possible to obtain good results for classification. Zhang et al [25] evaluated the performances of ResNet50 on 1131 images, reporting sensitivity, specificity, accuracy, and AUROC of 82%, 71.7%, 77%, and 0.846, respectively.

However, such results may not be easily translated on our institutional images: we can observe a general lack in sensitivity, even more so when networks are tested on BI-RADS 4 lesions only; specificity, too, is higher on BI-RADS 3 and BI-RADS 5 lesions for all CNNs. Hence, poor performances of CNNs trained on the BUSI dataset show that public images fail to capture the complexity of our institutional data, mainly due to the BI-RADS 4 amount that is challenging to classify. Hijab et al [19] found an accuracy of 0.97 and AUROC of 0.98 using VGG16 with fine-tuning on an institutional dataset of 1,300 images. Such results are higher than what we found, but no information is available on the BI-RADS classification distribution. Fujioka et al [26] found sensitivity, specificity, accuracy, and AUC of 0.958, 0.875, 0.925, 0.913, respectively, using a GoogleNet Inception V2, tested over 120 images, while Gu et al [27], using a VGG16 network trained on 4,149 ultrasound images found sensitivity, specificity, accuracy, and AUROC 0.888, 0.838, 0.864, and 0.913, respectively, on the external dataset. However, in the first case, only 43 of 120 cases were labeled as BI-RADS 4, while for the second study, the distribution of BI-RADS classification was not reported.

Li et al [18] using an updated version of ResNet50 found 0.733 sensitivity, 0.949 specificity, and 0.943 AUC, while Xiao et al [16] evaluated performances of ResNet50, InceptionV3, and Xception on 2,058 masses (1,370 benign and 688 malignant), finding sensitivity up to 0.891, specificity up to 0.774, accuracy up to 0.851 and AUC up to 0.91. However, for both studies, the BI-RADS classification distribution of the dataset is missing.

In particular, according to the BI-RADS reporting system, lesions classified with a BI-RADS score from 3 to 5 have an increasing probability of being malignant, starting from BI-RADS 3, whose probability should be lower than 2%, to BI-RADS 5, with a probability of malignancy of 95% or more. Management of BI-RADS 4 and 5 is quite straightforward, as tissue diagnosis through biopsy is recommended; on the other hand, despite the recommendation of short follow-up for BI-RADS 3, their management is somehow controversial, and up to 46% of BI-RADS 3 lesions may undergo biopsy [28]. In addition, the upper probability of malignancy for BI-RADS 3 (2%) might be underestimated for older women (up to 4.63 for women aged 80–90 years) [29].

Several studies demonstrated that the help of AI in classification can reduce the biopsy rate by 23% [30] or 27% [31]. In such a context, we exploited the potential of AI, since one interesting possibility of trained CNN is the capability to adjust the threshold for classification (*i.e.*, the probability value over which a lesion is considered as malignant). Despite overall good performances, in fact, the sensitivity and specificity of our networks would be too low to have those CNNs used in clinical practice as a support tool, but we can act on the threshold for classification, setting a proper value and evaluating again the performances of the networks.

Default is set to 50%, but it is possible to select other thresholds related to clinical value; if we consider lower thresholds than the default one at 50%, (*i.e.*, we consider a lesion as malignant if the probability computed by the network is higher than the threshold) we might improve sensitivity (lower FN rates) at a cost of decreased specificity (higher FP). In principle, every value from 0 to 1 can be used as a threshold; however, it is of utmost importance, when choosing a threshold different than the default value of 0.5, that the chosen level assumes clinical value. Since our aim was to correctly evaluate all positive lesions (*i.e.*, we do not want FN), we exploited this possibility with threshold adjustments, considering 1%, 2%, and 10% thresholds. In particular, a 2% threshold can be considered a “clinical threshold”: probably benign BI-RADS 3 lesions, in fact, should have a probability of malignancy lower than 2%, according to the BI-RADS system.

Despite the performances of ResNet50, which is not useful in this setting since it classifies all lesions as malignant, all other CNNs show very high sensitivity at the lowest thresholds (FN rate lower than 2%). As previously stated, the downside is a reduction in specificity, which, in some cases, may drop to 0%, making the predictions of no clinical interest. If we consider such a threshold of 2%, we found that Xception has a 0.983 sensitivity and 0.186 specificity, InceptionV3 shows 0.983 sensitivity and 0.169 specificity, while DenseNet121 has the same sensitivity and a slightly lower specificity of 0.153.

So, for these three networks, the FN rate was below 2%, and specificity results translated on a clinical scenario should have the potential to spare biopsy for 15.3–18.6% of patients with suspicious lesions. In those cases, CNNs would have suggested not to perform biopsy on lesions that were deemed worthy by radiologists, thus showing better performances. On the other hand, a 1.7% FN rate might be considered not acceptable, since, in principle, no cancer should be missed. However, when averaging the predictions of the three best-performing networks, we obtain a 100% sensitivity (*i.e.*, no cancer was missed), with a specificity of 10.2%.

Other studies present results of classification with CNNs, either custom-built or provided by software houses. Hayashida et al reported an AUC of 0.95, a sensitivity and specificity of 91.2% and 90.7%, respectively, on a test set evaluated by a CNN provided by a software house [32]. Qi et al reported the performances of two cascade networks that identify and classify suspicious lesions, with an accuracy of up to 90.1% that could be used for automatic diagnosis [33] while the EfficientDet algorithm, capable of providing precise segmentation and accurate classification simultaneously, was proposed by Du et al [34], with an accuracy of 92.6%. Hassanien et al reported a 91.7% accuracy using ConvNeXt on ultrasound real-time sequences, extracting features from each frame and estimating the malignancy score of each frame [35].

This study has some limitations. First, we assessed a limited number of images of the institutional dataset (*n* = 392); since contouring is a time-consuming and demanding task the number of images that have been used may be too small to represent a sufficient generalizable sample. However, this limitation may be overcome for future developments, since several studies demonstrated the feasibility of lesion recognition and boundary delimitation as a first step of lesion classification, thus allowing researchers to bypass the manual contouring process [36–38]. Gao et al, for example, reported an AUROC of 0.934 with a semi-supervised learning approach, in which a first network performed boundary definition, and then a VGG performed lesion classification [39]. Second, we used a single ultrasound device: the prospective evaluation of cases obtained with different devices could help in assessing the generalizability of our findings. Third, only a single expert breast radiologist contoured the images. However, in discrimination tasks, CNNs have demonstrated less variability than intrinsic interobserver variability of physicians assessing the presence of a disease [40]. Fourth, because of the limited number of images in our dataset, we did not perform fine-tuning on CNNs unfreezing some layers. We just added customized top layers for our tasks, as previously described, in order to obtain a simple configuration that can be reproducible, at the same time reducing the risk of overfitting. This is a possible cause for the lower performances of our trained CNNs compared to the results presented in the literature; on the other hand, the same configuration performed very well on the BUSI dataset, in some cases better than what is reported in other work. Hence, the main reason for such performances can be found in the high number of BI-RADS 4 (207 of 392, equals 52.8), namely lesions that are more difficult to learn from during training and to classify during tests.

To conclude, in this work, we evaluated the performances of different pre-trained networks in classifying suspicious breast lesions on ultrasound images. Our aim was to develop a design to exploit the potential of pre-trained networks, using all the information retrieved across the layers with just one layer to collect them, a dropout layer to avoid overfitting, and a final layer for classification, in order to create a highly reproducible experimental setup. We demonstrated that on a complex institutional dataset, the feasibility of classification after threshold adjustment had an FN rate lower than 2%, with the potential to spare biopsy in 15–18% of patients. In addition, when averaging predictions from the three best-performing networks, a 100% specificity with a TN rate of 10.2% was achieved. Combining the predictions of independent CNNs may have the potential to avoid FN, maintaining a level of specificity that could be useful in a clinical setting. Such performances should be further validated in prospective studies using images from different equipment, to assess the robustness of classification in order to use CNN predictions as support tools in clinical practice.

## Data Availability

The datasets used and/or analyzed during the current study are available from the corresponding author on reasonable request.

## Abbreviations

AI: Artificial intelligence
AUROC: Area under the receiver operating curve
BC: Breast cancer
BUSI: Breast ultrasound image
CNN: Convolutional neural network
FN: False negatives
FP: False positives
TN: True negatives
TP: True positives
